# Prevalence and phylogenetic analysis of tick-borne encephalitis virus (TBEV) in field-collected ticks (*Ixodes ricinus*) in southern Switzerland

**DOI:** 10.1186/1756-3305-7-443

**Published:** 2014-09-22

**Authors:** Nadia Rieille, Stéphane Bressanelli, Caio C M Freire, Séverine Arcioni, Lise Gern, Olivier Péter, Maarten J Voordouw

**Affiliations:** Central Institute of Valais Hospitals, Infectious diseases, Av Grand Champsec 86, Sion, Switzerland; Laboratory of Ecology and Evolution of Parasites, Institute of Biology, University of Neuchâtel, Rue Emile-Argand 11, 2000 Neuchâtel, Switzerland; Laboratoire de Virologie Moléculaire et Structurale, CNRS UPR3296, 1 avenue de la Terrasse, 91198 Gif-sur-Yvette cedex, France; Inter-institutional Grad Program on Bioinformatics, University of Sao Paulo, Matao Street 1010, Sao Paulo, Brazil; Central Institute of Valais Hospitals, Genetics, Av Grand Champsec 86, Sion, Switzerland; Laboratory of Eco-Epidemiology of Parasites, Institute of Biology, University of Neuchâtel, Rue Emile-Argand 11, 2000 Neuchâtel, Switzerland

**Keywords:** Envelope protein, Flavivirus, *Ixodes ricinus*, Switzerland, Tick, Tick-borne encephalitis virus, Tick-borne disease

## Abstract

**Background:**

Tick-borne encephalitis is the most common tick-borne viral infection in Europe with 3,000 human cases reported each year. In Western Europe, the castor bean tick, *Ixodes ricinus*, is the principal vector of the tick-borne encephalitis virus (TBEV). TBEV appears to be spreading geographically and was recently detected for the first time in Canton Valais in the southern part of Switzerland. The purpose of the present study was to survey the *I. ricinus* tick populations of Canton Valais for TBEV.

**Methods:**

We collected a total of 19,331 *I. ricinus* ticks at 45 different sites in Canton Valais between 2010 and 2013. Ticks were processed in pools and tested for TBEV using reverse transcription quantitative PCR. The *NS5* gene and the *envelope* gene of the TBEV isolates were partially sequenced for phylogenetic analysis.

**Results:**

TBEV was detected in tick populations at six of the 45 sites. These six sites were all located in a 33 km transect along the Rhône River. TBEV was detected in two sites for three of the four years of the study showing the temporal persistence of the pathogen. Prevalence of TBEV in the six positive sites ranged from 0.16% to 11.11%. Phylogenetic analysis found that all TBEV isolates from Canton Valais belonged to the European subtype. Genetic analysis found two distinct lineages of TBEV suggesting that Canton Valais experienced two independent colonization events.

**Conclusions:**

TBEV appears to be well established at certain locations in Canton Valais.

**Electronic supplementary material:**

The online version of this article (doi:10.1186/1756-3305-7-443) contains supplementary material, which is available to authorized users.

## Background

Tick-borne encephalitis is the most important viral tick-borne disease in Europe [1]. The causative pathogen is a single-stranded RNA virus that belongs to the tick-borne flavivirus group (genus *Flavivirus*, family *Flaviviridae*). This family of arboviruses includes dengue and yellow fever virus. The tick-borne encephalitis virus (TBEV) exhibits genetic variation across its geographic range and three subtypes of TBEV are currently recognized: European (TBEV-Eu), Siberian (TBEV-Sib), and Far Eastern (TBEV-FEa) [2–4]. All three TBEV subtypes attack the central nervous system of the host with potentially fatal outcomes in humans. The European subtype is the least virulent subtype and is responsible for all human cases of TBE in Western Europe.

TBEV is a zoonotic pathogen that is maintained in nature by cycling between competent reservoir hosts and a tick vector. The most important vertebrate reservoir hosts for the virus are small mammals such as rodents [5]. In Western Europe, the main vector of TBEV is the castor bean tick, *Ixodes ricinus*. This tick has three stages: larva, nymph, and adult and each stage takes a single blood meal from a different host to complete its development to the next stage. Larvae are generally uninfected because transovarial (vertical) transmission of TBEV is rare [6, 7]. Larvae (or nymphs) acquire TBEV during the blood meal and maintain the infection after molting into the nymphal stage (or adult stage) in a process called transstadial transmission. Once infected, ticks carry the virus for life [8]. From an epidemiological perspective, infected nymphs are the most important stage because they are much more numerous than infected adult ticks.

The proportion (or prevalence) of TBEV-infected ticks in populations of *I. ricinus* is often low. In European areas where TBEV is endemic, the prevalence of the virus in *I. ricinus* populations varies from 0.1 to 5.0% [9]. At a spatial scale, the distribution of TBEV-infected ticks and reservoir hosts is highly patchy and these patches are often referred to as foci [10, 11]. There is substantial temporal variation in the prevalence of TBEV and newly established foci will not necessarily persist through time [12]. Surveillance of TBEV foci is therefore necessary to establish if the risk of infection remains or has disappeared.

Currently, TBE is known to be endemic in Eurasia from Central Europe to Japan [13]. In Europe, 3,000 human cases are reported each year and this number rises to 10,000 cases per year if Russia is included [14]. In recent years, the human case load of TBE appears to be increasing in regions where it had not been previously observed such as Scandinavia [15–18], and France [19] as well as in TBE-endemic areas such as eastern Europe [20]. This increasing human caseload may be explained by several factors including improved diagnosis, climate change (in Northern Europe), and socio-economical changes (in Eastern Europe) [20, 21]. In addition, phylogeographic approaches have been used to study the evolutionary history and dispersal of TBEV in Eurasia [22–24]. At the continental scale, TBEV originated in Eastern Europe before spreading westward across the European continent over a period of 2,500 years [22, 25]. Studies at finer geographic scales have shown a variety of dispersal patterns associated with both biogenic and anthropogenic factors [23, 26].

In Switzerland, TBE in humans was first described in 1969 via serology [27, 28]. In the following years, TBEV was identified in tick populations from the northeastern and central part of the country [29–32]. During the last decade, the virus and the disease appear to have spread to the western part of Switzerland [33–35]. Phylogenetic analysis of the Swiss isolates found several distinct lineages of the European subtype suggesting multiple introductions of TBEV [33, 34]. To date, little is known about the prevalence of TBEV in the southern part of Switzerland including Canton Valais. In 2009, a national survey collected more than 62,000 ticks from all over Switzerland and screened them for TBEV [33]. This survey detected TBEV in 38 areas in Switzerland including two areas in Canton Valais: Raron and Salgesch [33]. This was the first time that TBEV had been reported in the southern part of Switzerland.

The present study further investigates the prevalence of TBEV in *I. ricinus* tick populations in Canton Valais. The first objective of the study was to confirm the persistence of TBEV in Raron and Salgesch, the two risk areas previously identified by the national survey. The second objective was to test other tick populations in Canton Valais for the presence of TBEV. We also conducted a phylogenetic analysis to determine the origin and relatedness of the Valais TBEV isolates. The present study allows us to evaluate the risk of TBE in Canton Valais.

## Methods

### Collection of ticks in the field

The fieldwork was conducted between May 2010 and June 2013. We surveyed the two sites in Canton Valais that had previously been identified as TBEV foci by the national survey: Raron (2.7 ha) and Salgesch (41 ha) [33]. Ticks were also collected from 43 additional sites distributed throughout Canton Valais (see Additional file 1). The dominant criteria for selecting these sites were percent forest cover and road access. Most sites had an elevation between 390 and 1600 m and the habitat consisted of forest and bushes. We collected ticks by dragging a white cotton towel (surface of 1.0 m^2^) over the vegetation. The towel was inspected for ticks every 10 m and ticks were identified to the species level using the key by Cotty (1985) [36]. All *I. ricinus* ticks were brought to the laboratory and stored in tubes at -80°C until further analysis.

### Tick pooling and RNA extraction

Ticks were screened for TBEV using the methods described in Gäumann *et al*. (2010) [33]. The 19,331 ticks were grouped in 1,033 pools of 10**–**20 adults or 50 nymphs to increase the efficiency of the screening procedure. Pools of ticks were placed in individual 2 ml micro tubes with 250 μl of PBS (0.1 M, pH 7.2) and stored at 2**–**8°C. Ticks were crushed by shaking them in the presence of a stainless steel bead (7 mm diameter) at 50 Hz for 10 minutes using the TissueLyser system (Qiagen). The lysate volume was adjusted with PBS to a final volume of 3.5 ml in a larger tube. After centrifugation (2 min at 960 g), 650 μl of supernatant from each sample was used for RNA extraction, which was performed using the AmpliPrep COBAS (Roche, Rotkreuz, Switzerland) and Cobas AmpliPrep Total Nucleic Acid Isolation (TNAi) kits. The RNA was eluted in a final volume of 75 μl of elution buffer consisting of phosphate buffer, sodium chloride and sodium azide.

### One-step reverse transcription quantitative PCR (RT-qPCR)

We used reverse transcription quantitative PCR (RT-qPCR) to test whether the 1,033 tick pools were infected with TBEV. The RT-qPCR targeted a fragment of the TBEV *envelope* (*E*) gene using the primers and probes described by Gäumann *et al*. (2010) [33]. We amplified the 5’ non-coding region of the Mengo virus as an internal control for each of the 1,033 RT-qPCR reactions. An extract of the TBEV vaccine (Baxter, Switzerland) was used as the positive control and distilled water was used as the negative control. Samples were processed in 46 experimental blocks with 20 to 45 samples per block. Each experimental block contained one positive control and one negative control for every five samples. Experimental blocks were considered valid if the internal controls and the positive controls were detected. Samples were considered positive when TBEV was detected before 40 cycles with a threshold of 0.05 fluorescence units. All TBEV-positive samples were retested twice and all such samples tested positive three times. RT-qPCR was performed using the Thermo scientific Verso 1-step QRT-PCR plus Rox kit (Thermo scientific, Surrey, UK).

The RT-qPCR reaction for each pooled tick sample contained 5 μl of RNA template and 20 μl of master mix. The 20 μl master mix contained 12.5 μl of 1-Step QPCR Mix, 1.25 μl of QRTase Enhancer, 0.25 μl of Verso Enzyme Mix, 4 μl of primers (0.5 μM), 2 μl of probes (0.2 μM), and 0.02 μl of Mengo virus cDNA. The 4 μl primers solution contained 1 μl of each of the forward (tbeE-F6) and reverse (tbeE-R2) primers targeting the terminal part of the *E* gene of TBEV and 1 μl of each of the forward (Mengo-F1) and reverse (Mengo-R1) primers targeting the 5’ non-coding region of the Mengo virus (see Additional file 2). The 2 μl probes solution contained 1 μl of each of the TBEV (TBEE-P4) and the Mengo virus (Mengo-P1) probes (see Additional file 2). Cycling conditions were as follows: reverse transcription at 50°C for 15 min, an initial PCR activation step at 95°C for 15 min, and 45 cycles of 95°C for 15 sec and 60°C for 1 min. A Corbett research Rotor-Gene RG-3000 (Switzerland) was used for amplification.

### Statistical analysis

Screening pools of ticks is a time-efficient and commonly used method to determine TBEV prevalence in ticks [15, 33, 34, 37–41]. One disadvantage of this approach is the loss of information; it is no longer possible to determine whether a positive pool contained one or more infected ticks. The minimum infection rate assumes that a positive pool contains a single infected tick [42]. All MIR estimates will be reported as percentages in the results. The numbers of ticks collected were quite low at some sites and we therefore performed power analyses to determine the probability of detecting a prevalence of TBEV greater than 1.0%.

### Genetic analysis of TBEV sequences

We used genetic analysis to compare our TBEV sequences from Canton Valais to other TBEV sequences from Switzerland. We partially sequenced each of two genes of TBEV: the *NS5* gene and the *E* gene. The *NS5* gene codes for a highly conserved, non-structural protein with methyltransferase and RNA-dependent RNA polymerase activities [43]. The *E* gene codes for a protein that mediates the binding of the virus to the host cells and subsequent membrane fusion [43, 44]. Numerous studies have used the *NS5* gene [32, 45–47] and the *E* gene [2, 46, 48–51] to study the genetics and evolution of TBEV in Europe and Switzerland [34, 52]. We therefore analyzed our partial sequences of the *NS5* and *E* genes to facilitate comparison between our TBEV sequences from Canton Valais and the rest of Switzerland and Europe.

### Sequencing protocols

All TBEV-positive samples in the RT-qPCR were sequenced with respect to part of the *NS5* gene (212 bp) and part of the *E* gene (752 bp) using the primers designed by Puchhammer-Stöckl *et al*. (1995) [53] and Gäumann *et al.* (2011) [52]. The *NS5* gene sequences for the 2010 and 2011 samples were obtained using an in-house sequencing protocol. The *NS5* gene amplicons for the 2013 samples and the *E* gene amplicons for all the samples (2010, 2011, and 2013) were sequenced by Microsynth. All chromatographs were checked for the accuracy of the base calls and the overall quality of the peak shape using the 4peaks software package (version 1.7.2).

### Phylogenetic analysis

Phylogenetic analysis included a total of 80 nucleotide sequences: 24 sequences from the present study and 56 reference sequences from the NCBI GenBank database (see Additional file 3). The Omsk hemorrhagic fever virus [Genbank assession number AY323489], a closely related flavivirus, was chosen as the outgroup. The sequences of eight European isolates, three Siberian isolates, and three Far Eastern isolates were used to confirm the European subtype classification of the isolates found in Canton Valais. The genetic analyses of the *NS5* gene and the *E* gene contained 17 and 24 additional gene sequences, respectively (see Additional file 3).

DNA sequences were aligned using the default settings in Clustal Omega [54]. The alignments were concatenated and absent sequences were replaced by missing data. We used the Genetic Algorithm for Rapid Likelihood Inference (GARLI version 2.0) software package [55] for phylogenetic analysis. Phylogenetic trees were generated by a stochastic algorithm, which uses maximum likelihood (ML) to simultaneously search for the best tree topology, branch lengths, and nucleotide substitution model parameters. We used a nucleotide substitution model based on a general time reversible model [56], where variation in the nucleotide substitution rate follows the gamma distribution (Γ) [57] and a proportion of invariable sites (I). Support for the topology was obtained after 1,000 non-parametric bootstrap replicates with GARLI. The bootstrap trees were summarized into one consensus tree using Dendropy v3.10.1 [58]. Our approach of using missing data is consistent with ML methods where phylogenetic accuracy can be improved with incomplete data [59].

To evaluate patterns of selection on the partial sequences of the *NS5* gene and the *E* gene, we estimated the difference (ω = dN–dS) between the non-synonymous (dN) and synonymous [60] substitution rates per codon site, using the single likelihood ancestor counting (SLAC) algorithm with HyPhy v2.11 [61]. Values of ω greater than zero suggest directional selection while values below zero suggest purifying selection. We used an ANOVA to test whether there were significant differences in the pattern of selection among the different functional regions of the *E* gene.

### 3D Structural analysis of the envelope protein

We used 3D structural analysis to study the shape of the Envelope (E) protein of TBEV. A previous study on the E protein of TBEV had determined the X-ray crystal structure of the N-terminal ectodomain (residues 1-395) [62]. In the present study, all the substitutions were located in the C-terminal part (see Results) for which no atomic level data is available. We therefore used the known 3D structure of the E protein of the dengue virus (Protein data Bank accession code 3 J27) to model the E protein of TBEV. The amino acid similarity among the flavivirus envelope proteins is 40% [63, 64], which ensures that the homology model is accurate [65]. We used the HHPRED software program [66] from the MPI Bioinformatics Toolkit web service [67] to align amino acid sequences and to predict the three-dimensional structure of the full-length TBEV envelope protein. We validated our approach by confirming that the 395 N-terminal residues of our homology model gave a good match to the known structure of the TBEV ectodomain (PDB 1SVB).

## Results

### Ticks

**Figure 1:**
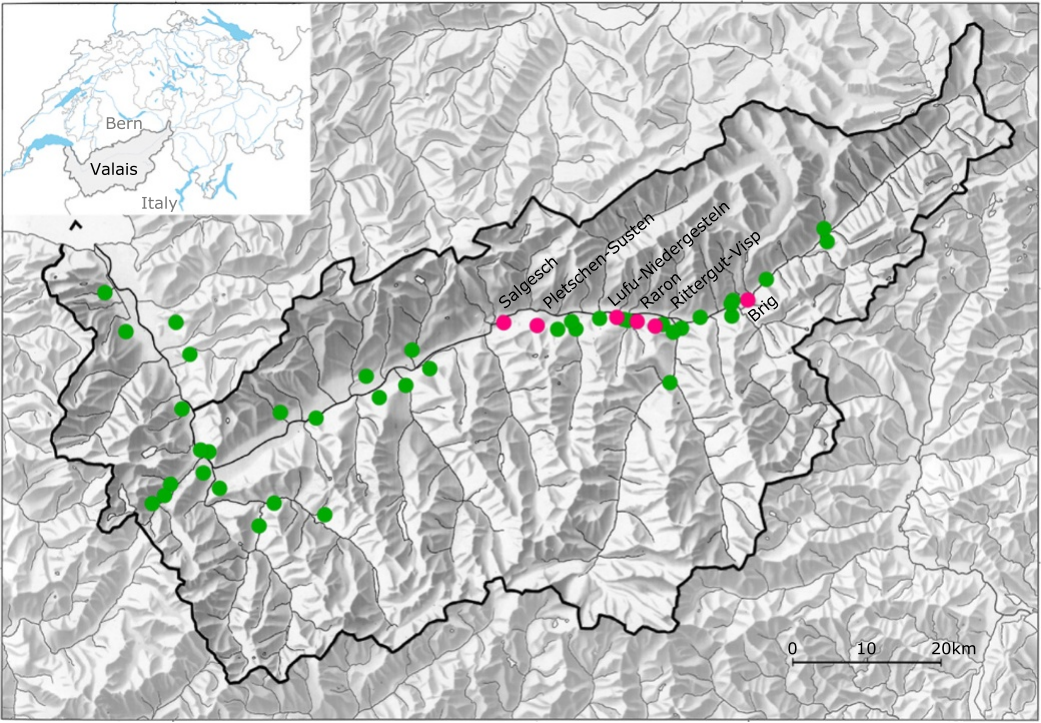
**Map of the 45 sites in Canton Valais, Switzerland sampled for** ***Ixodes ricinus*** **ticks.** The six sites where tick populations tested positive for TBEV are named and shown in red. The 39 sites where tick populations tested negative for TBEV are shown in green. The map was made using the QGIS 1.8.0 Lisboa software.

Over the four years of the study, a total of 19,331 *I. ricinus* ticks were collected in Canton Valais: 11,142 nymphs (57.64%), 7,976 adults (41.26%) and 213 larvae (1.10%; Additional file 4). Of the 45 sites, six were found positive for TBEV including the two sites, Raron and Salgesch, which had been previously identified as TBEV foci in 2009 by Gäumann *et al.* (2010) [33]. All positive sites were located in a 33 km long transect along the Rhône River (Figure 1). In addition to the 19,331 *I. ricinus* ticks, we sampled 99 *Haemaphysalis punctata* and 66 *Dermacentor marginatus* ticks but none of these ticks tested positive for TBEV.

In 2010, 6,507 *I. ricinus* were collected from 20 sites: 3,199 adults (49.16%), 3,306 nymphs (50.81%), and 2 larvae (0.03%). The presence of TBEV in ticks was confirmed that year at both Raron (n = 1,749 ticks) and Salgesch (n = 489 ticks). The minimum infection rate [42] was 0.74% (95% confidence limits (CL) = 0.39**–**1.27%) at Raron and 0.20% (95% CL = 0.01**–**1.13%) at Salgesch (Table 1). The remaining 4,269 ticks collected at the other 18 sites in Valais were all negative for TBEV.

**Table 1:** **The minimum infection rate** [42] **of** ***Ixodes ricinus*** **ticks at the TBEV foci**

		Number of ticks sampled			Positive pools/total pools			Adults			Nymphs			Mix		
Site	Year	Adult	Nymph	Larva	Adult	Nymph	Mix ^a^	MIR	LL	UL	MIR	LL	UL	MIR	LL	UL
Raron	2010	875	872	2	11/135	0/45	2/21	1.26	0.63	2.24	0.00	0.00	0.42	0.74	0.39	1.27
Salgesch	2010	271	218	0	0/11	1/6	0/0	0.00	0.00	1.35	0.46	0.01	2.53	0.20	0.01	1.13
Lufu-Niedergesteln	2011	38	66	0	1/2	1/2	0/0	2.63	0.07	13.81	1.52	0.04	8.16	1.92	0.23	6.78
Raron	2011	156	124	0	2/30	0/8	0/0	1.28	0.15	4.55	0.00	0.00	2.93	0.71	0.08	2.56
Rittergut-Visp	2011	73	10	0	2/6	0/1	0/0	2.74	0.33	9.55	0.00	0.00	30.85	2.41	0.29	8.44
Salgesch	2011	320	1012	0	1/38	2/23	0/0	0.31	0.01	1.73	0.2	0.02	0.72	0.23	0.05	0.66
Brig	2013	397	213	1	1/19	0/4	0/1	0.25	0.01	1.40	0.00	0.00	1.72	0.16	0.01	0.91
Pletschen_Susten	2013	7	2	0	0/0	0/0	1/1	0.00	0.00	40.96	0.00	0.00	84.19	11.11	0.28	48.25
Raron	2013	261	239	0	0/14	0/6	1/1	0.00	0.00	1.40	0.00	0.00	1.53	0.20	0.01	1.11
Salgesch	2013	222	434	6	1/14	1/9	0/1	0.45	0.01	2.49	0.23	0.01	1.28	0.30	0.04	1.09
Total		2620	3190	9	19/269	5/104	4/25	0.73	0.44	1.13	0.16	0.05	0.36	0.48	0.32	0.7

LL = lower 95% confidence limit.

UL = upper 95% confidence limit.

^a^mixture of adults, nymphs and larvea.

For each of the ten combinations of site and year, the tick sample sizes are shown for each stage (adult, nymph, larva). The proportion of TBEV-positive pools is shown for pools of three different stage compositions: adults, nymphs, and mixture (adults, nymphs, larvae). The MIR is shown separately for adults, nymphs, and mixture (adults, nymphs, larvae). The MIR and the 95% confidence limits are expressed as a percent.

In 2011, 6,804 ticks were collected from 19 sites: 2,262 adults (33.25%), 4,535 nymphs (66.65%), and 7 larvae (0.10%). The tick populations at Raron and Salgesch remained infected with the virus. The MIR was 0.71% (95% CL = 0.08**–**2.56%) at Raron and 0.23% (95% CL = 0.05**–**0.66%) at Salgesch. TBEV-infected ticks were detected at two additional sites this year: Rittergut-Visp (MIR = 2.41%; 95% CL = 0.29**–**8.44%) and Lufu-Niedergesteln (MIR = 1.92%; 95% CL = 0.23**–**6.78%; Table 1).

In 2012, 2,813 ticks were collected from eight sites. None of the ticks tested positive for TBEV including the ticks from the Raron (n = 718 ticks) and Salgesch (n = 467 ticks) sites. Under the assumption that the minimum infection rate was at least 1%, the 2012 sampling effort at both sites had a power of more than 99% to detect at least one TBEV-infected tick (see Additional file 4).

In 2013, 3,207 ticks were collected from nine sites: 1,035 adults (32.27%), 1974 nymphs (61.55%), and 198 larvae (6.17%). The MIR was 0.20% (95% CL = 0.01**–**1.11%) in Raron and 0.30% (95% CL = 0.04**–**1.09%) in Salgesch. TBEV was detected for the first time in ticks collected at Brig and Pletschen-Susten (Table 1) with a MIR of 0.16% (CL = 0.01**–**0.91%) and 11.11% (CL = 0.28**–**48.25%), respectively.

Of the 28 pools of ticks that tested positive for TBEV between 2010 and 2013, 19 contained adults, 5 contained nymphs and 4 contained a mix of adults and nymphs (Table 1). After summing all the ticks across the TBEV-positive sites, the MIR of adult ticks (0.73%; 95% CL = 0.44–1.13%) was 4.6 times higher than that of the nymphal ticks (0.16%; 95% CL = 0.05–0.36%) and this difference was statistically significant (χ^2^ = 9.96, df = 1, p < 0.002; Table 1).

### Genetic analysis

Of the 28 pools of ticks that tested positive for TBEV, 25 pools were successfully sequenced for part of the *NS5* gene and 21 pools were successfully sequenced for the terminal part of the *E* gene. A total of 46 sequences were submitted to the NCBI GenBank database (see Additional file 5 for accession numbers).

The 25 partial DNA sequences of the *NS5* gene included two different variants. One variant contained 24 DNA sequences from five different sites (Raron, Salgesch, Niedergesteln, Susten, Visp), whereas the other variant, which had 5 nucleotide substitutions, consisted of a single sequence from the 2013 Brig site. DNA sequence variability in the *NS5* gene was therefore low (2.35% = 5 variable nucleotides/212 total nucleotides). One of the five nucleotide substitutions (20.0%) was non-synonymous and occurred at position number 302 in the *NS5* gene.

**Figure 2:**
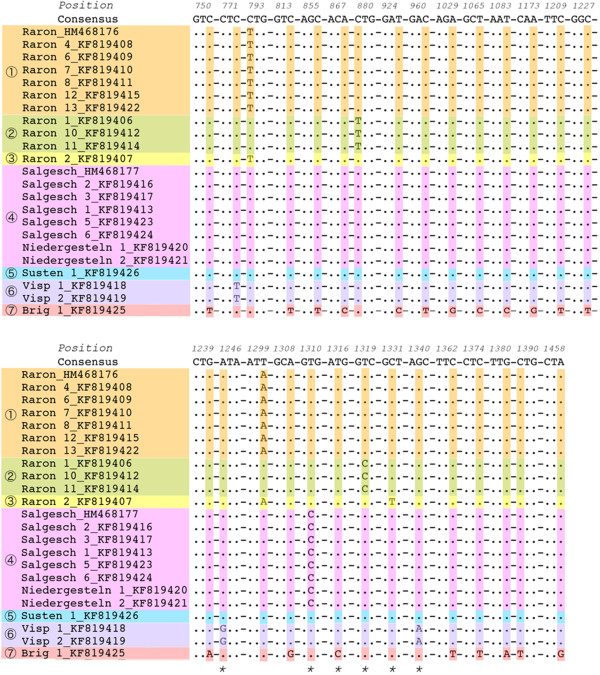
**The TBEV isolates from Canton Valais had seven different variants of the terminal** ***envelope*** **gene.** The length of the partial sequence of the *envelope (E)* gene was 752 bp but only the 29 codons with a nucleotide substitution (highlighted in color) and their position on the *E gene* are shown. Non-synonymous substitutions are indicated with an asterisk. Sequences were aligned using EMBOSS Showalign (http://emboss.ch.embnet.org/Pise/showalign.html).

The 21 partial DNA sequences of the *E* gene included seven different variants. The Raron site had three *E* gene variants whereas all other sites had a single variant. The *E* gene variant in Salgesch was the same as the variant in Lufu-Pletschen. The terminal part of the *E* gene had 29 nucleotide substitutions and the DNA sequence variability was low (3.85% = 29 variable nucleotides/752 total nucleotides). Six of the 29 substitutions (20.7%) were non-synonymous and all these mutations were assembled on a 94 bp fragment of the *E* gene (Figure 2). Twenty of the 29 nucleotide substitutions were only found in the Brig isolate suggesting that this variant was quite different from the other variants collected in Valais.

Comparison of the synonymous [60] and non-synonymous (dN) substitution rates found that dS > dN at all amino acid positions in the partial sequences of both the *NS5* gene and the *E* gene (data not shown). This result suggests that both the *NS5* gene and the *E* gene are under purifying selection. For the subset of the 60 Swiss TBEV isolates, the ANOVA found no significant differences in the pattern of selection among the five domains of the Envelope protein (F_4, 243_ = 0.82, p = 0.514).

### Phylogenetic analysis

**Figure 3:**
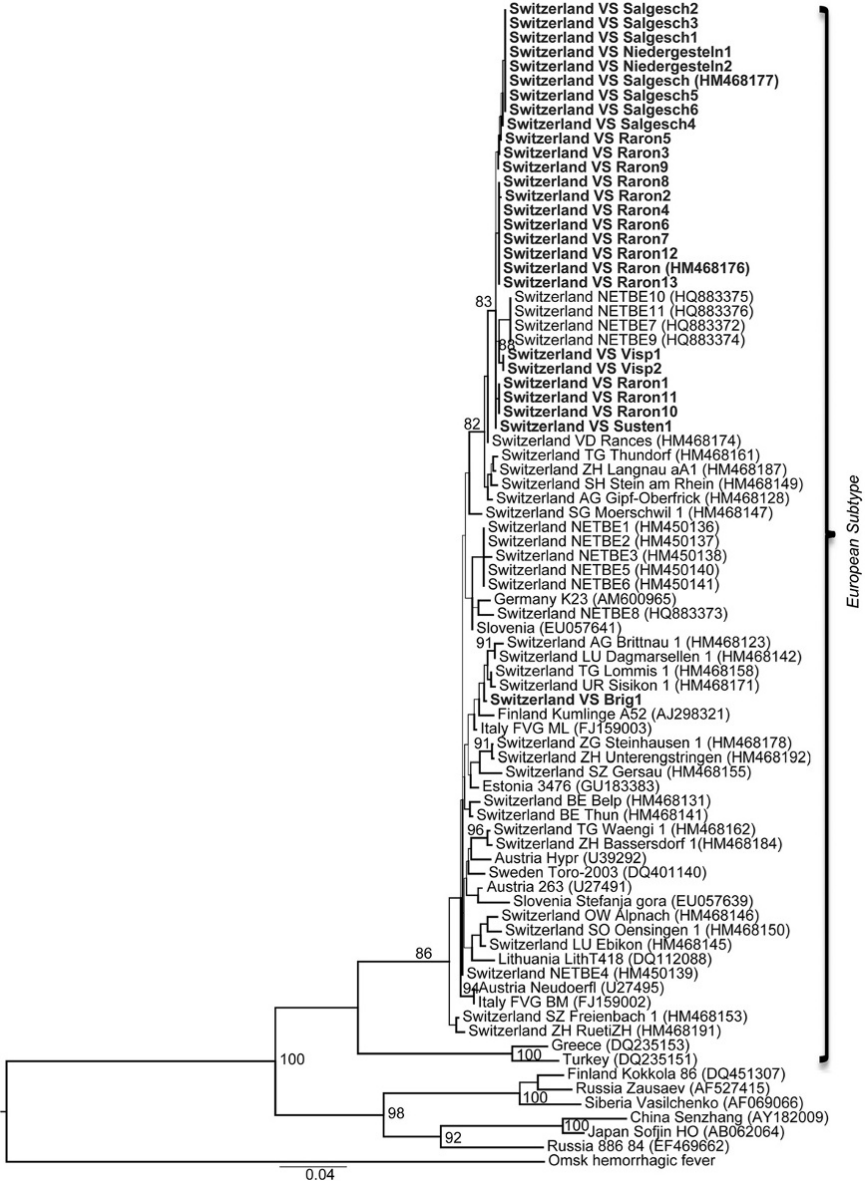
**Phylogenetic tree of the TBEV isolates from Canton Valais.** The sequences of the TBEV isolates were based on concatenation of the *NS5* gene (212 bp) and the *envelope* gene (752 bp). The Omsk hemorrhagic fever virus was used as an out-group to root the tree. The TBEV sequences from Canton Valais are shown in bold. GenBank accession numbers are indicated and bootstrap values ≥ 80% are shown. The scale bar corresponds to 0.04 nucleotides per site.

In this study, all TBEV isolates from Canton Valais belonged to the European subtype. The DNA sequences of the seven strains from Canton Valais were 98.24 to 97.00% similar to the reference strain of the European subtype (Neudoerlf; Genbank = U27495). In contrast, the similarity with the reference strains of the Far Eastern (Sofjin; Genbank = AB062064) and the Siberian (Vasilenko; Genbank = AF069066) subtypes ranged between 83.50% and 87.81%. All isolates from Canton Valais were closely related to each other, except the isolate from Brig (C20m), and they were closely related to other Swiss TBEV isolates obtained from previous studies [32, 34, 52] (Figure 3).

### 3D Structural analysis

**Figure 4:**
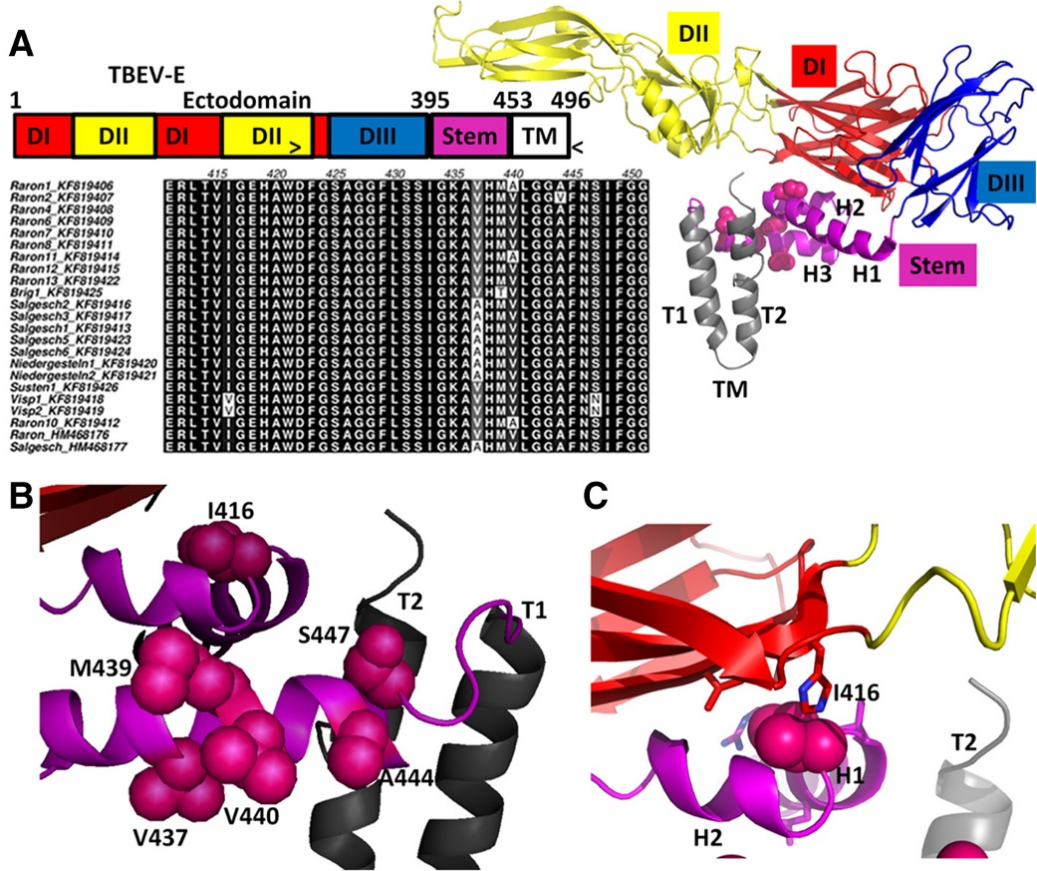
**Locations of the mutations in the Envelope protein of TBEV are shown. (A)** Left, top: the primary structure of the TBEV envelope protein and its organization into the ectodomain, stem, and trans-membrane (TM) region. The region encompassing amino acid residues 249-496 (indicated by “>” and “<”) corresponds to the terminal part of the *envelope* gene that was sequenced in this study. All non-synonymous substitutions in the partial sequences of the Canton Valais TBEV isolates mapped to the stem region of the Envelope protein. **(A)** Left, bottom: Alignment of the Envelope protein amino acid sequences of the 21 Canton Valais TBEV isolates reported in this study and two reference sequences (Raron_HM468176 and Salgesch_HM468177). Amino acid substitutions were found at positions 416, 437, 439, 440, 444 and 447. Right: the three-dimensional structure of a single TBEV E molecule based on the homology model of the dengue virus E molecule (PDB 3 J27). The N-terminal ectodomain is attached to the stem and consists of three domains: central domain (D1; in red), the fusion domain (D11; in yellow), and the lateral domain (DIII; in blue). The stem consists of three helices (H1, H2, and H3; in magenta) and the side chains of the amino acid substitutions are shown (pink spheres). The two trans-membrane helices (T1 and T2; in gray) are inserted in the viral membrane. **(B)** Close-up of the variable residues in the H3 helix of the stem. The mutated residues are labeled. **(C)** Close-up of the mutant I416 residue, which projects into a hydrophobic pocket (residues lining this pocket are displayed as sticks).

The 3D structure of the full-length Envelope (E) protein of TBEV is currently unknown. We therefore used the E protein of the dengue virus [68] to create a homology model of the E protein of TBEV (Figure 4). The flavivirus E protein (~495 amino acids) consists of a large N-terminal ectodomain (~395 amino acids) [62] and a C-terminal trans-membrane region (anchor) connected by the stem region (Figure 4A). This stem region (colored magenta in Figure 4) consists of three helices (H1, H2, and H3) that lie flat on the viral membrane (Figure 4A). Our partial DNA sequences of the *E* gene only encode a portion of the E protein from amino acid residues 249 to 496 (C-terminus). This portion of the E protein includes part of the ectodomain, the stem and the trans-membrane region. All non-synonymous substitutions in the partial *E* gene sequences of the Canton Valais isolates mapped to the stem region. Most of the amino acid substitutions (e.g. residues 437, 440 and 444) were located in the H3 helix (Figure 4B). One amino acid substitution (residue 416) was located at the break between the H1 and H2 helices (Figure 4C).

## Discussion

Tick-borne encephalitis virus (TBEV) was detected in questing *Ixodes ricinus* ticks in 2010, 2011 and 2013 at Raron and Salgesch in Canton Valais, confirming the temporal persistence of these two TBEV foci since their discovery in 2009 [33]. The virus was not detected in 2012 at either site but this could be explained by the smaller sample sizes of ticks collected that year (n = 718 ticks for Raron and n = 467 ticks for Salgesch). Our power analysis of the 2012 data showed that we had a 75% chance of detecting TBEV-positive pools if the 2012 MIR was equal to the 2013 MIR (0.20%). A previous field study spanning ten years in Latvia found that the prevalence of TBEV in *I. ricinus* ticks was characterized by high temporal variability [69, 70]. In addition to confirming the TBEV-positive status of Raron and Salgesch, this study found four new TBEV foci. All six TBEV foci were located in a 33 km long transect along the Rhône River (Figure 1). Repeated detection of the virus at Raron and Salgesch and the discovery of four new TBEV foci indicate that this part of Canton Valais is a risk area for TBEV.

Altitude can play an important role in the distribution of *I. ricinus* and thus tick-borne infections. In the present study, the elevation of the six TBEV foci ranged between 570 m and 980 m above sea level and none of the eight sites above 1,000 meters tested positive for TBEV. Studies in other mountain regions have found TBEV-infected ticks or goats at higher elevations. In the Czech Republic, TBEV-infected *I. ricinus* populations were found up to 1,100 meters [71]. In the Austrian Alps, cases of human TBE caused by non-pasteurized goat milk have occurred at elevations above 1,500 meters [72]. In Canton Valais, stable populations of *I. ricinus* have been found at 1,450 meters [73]. Taken together, these studies suggest that TBEV could spread to higher elevation tick populations in the future. Epidemiological models incorporating climate change project that TBEV will be found at higher altitudes and latitudes in Europe in the coming decades [12] although these same models project that TBEV will be extirpated from most regions of Switzerland by 2020 [74].

Detection of TBEV-infected ticks at Rittergut-Visp in 2011 was linked with the first autochthonous human case of TBE described in Canton Valais [75]. In contrast, a tick collection site (Oberi Albe-Visp), which was only 200 meters from the exact location where the TBE patient was bitten, tested negative for TBEV in that same year. This example shows the spatio-temporal patchiness of TBEV in tick populations and also shows the difficulty of using TBEV prevalence in ticks to predict the risk of TBE for humans. Similarly, previous studies in Poland, Germany and Switzerland found that tick sampling could not assure virus detection in known endemic foci [34, 76, 77].

Temporal persistence of TBEV-positive ticks in a given location remains the gold standard for defining TBEV foci. However, the generally low prevalence of TBEV in tick populations also means that this gold standard is time-consuming and inefficient. For this reason, numerous field studies have used serology of vertebrate hosts to identify TBEV foci [78–84]. As vertebrate hosts can feed many ticks, they effectively amplify the TBEV signal in a given area. In the present study, the TBEV foci discovered at Pletschen-Susten and Brig in 2013 were predicted from an immunological survey of sera obtained from local populations of domestic goats (Rieille, in preparation). These preliminary results confirm that using vertebrate hosts as sentinels is an efficient method of detecting new TBEV foci [80, 85, 86].

In this study, a total of 19,331 *I. ricinus* ticks were collected over four consecutive years and processed in pools, of which 2.7% (28/1,033) tested positive for TBEV (see Additional file 4). The prevalence of TBEV in the tick populations of Canton Valais (Table 1) was similar to previous studies in Europe [15, 47, 87, 88] and Switzerland [32–34, 40, 89]. In these other studies, the prevalence of TBEV in free-living ticks ranged from 0.1 to 5.0% [9] The high prevalence found at Pletschen-Susten (11.11%) was based on a small sample size (n = 9 ticks). A few other studies have found unusually high prevalence of TBEV in ticks such as 14.3% in an endemic area of central Switzerland [90] or 14.0% and 26.6% in Slovakia and Latvia, respectively [70, 91]. Across the ten TBEV-positive combinations of site and year (Table 1), the MIR of adult ticks was 4.6 times higher than that of nymphs and this difference was statistically significant. We expected adult ticks to have a higher infection prevalence than nymphal ticks because they have taken twice as many blood meals and are therefore twice as likely to have encountered the virus [8]. Other studies have found that the prevalence of TBEV is higher in nymphs than in adult ticks [32, 90].

Phylogenetic analyses of the partial sequences of the *NS5* gene and the *E* gene found that all TBEV strains from Canton Valais belonged to the European subtype and were closely related to other Swiss isolates (Figure 3). The isolate from Brig appeared to belong to a different lineage. The presence of two TBEV lineages in Canton Valais suggests that TBEV emerged at Brig independently from the other TBEV foci. We currently do not know when and how TBEV reached Canton Valais. Canton Valais is surrounded by high mountain ranges to the north and south (2,000 to 3,000 meters above sea level) that are difficult to cross for the relevant reservoir hosts (i.e. rodents). This geological isolation may explain why this Canton has remained TBEV-free for so long even though the virus is endemic in most of Switzerland. There is some evidence that birds can disperse TBEV-infected ticks during migration [92–94] and that ticks can persist when transported to new habitats by birds [95]. Cervids are another plausible dispersal host for TBEV because they are both highly mobile and frequently parasitized by ticks. A number of studies have shown that cervids such as roe deer play an important role in the epidemiology of TBE [80, 96]. These animals could bring the virus into Canton Valais by following low elevation wildlife corridors. Thus both birds and cervids could have played an important role in bringing TBEV to Canton Valais.

Anthropogenic activity such as road construction [23] and landscape features such as river systems [26] may also play a role in the dispersal of TBEV. Transport tunnels that cross the surrounding mountain ranges connect Canton Valais to TBEV-endemic areas. For example, the Lötschberg Tunnel, which was opened in June 2007, is a 34.5 km railway tunnel that connects Raron in Canton Valais to Frutigen in Canton Bern, where TBEV is endemic. The Simplon Tunnel is a 19.8 km auto tunnel that connects Brig in Canton Valais to Iselle in northern Italy. A study on this tunnel demonstrated the presence of house mice (*Mus domesticus*), and thus another route by which TBEV could enter Canton Valais [97]. In the present study, we could not demonstrate that strains from Canton Valais were genetically more similar to strains from Canton Bern (HM468131; HM468141), strains from Italy (FJ159003; FJ159002) or strains from other geographic locations represented in our analysis. Future studies examining more sequences from surrounding areas may help clarify the origins of the TBEV strains in Canton Valais.

Genetic variability was low for both the *NS5* and the *E* gene but the latter was slightly better for distinguishing variants. Comparison of the non-synonymous and synonymous substitution rates found that numerous sites in the *NS5* gene and the *E* gene were under purifying selection. These results are in agreement with other studies showing that the TBEV genome is under purifying selection [26, 51]. However, we found no evidence that the pattern of selection varied among the different domains of the Envelope protein.

The Envelope protein of TBEV is composed of two regions: the so-called ectodomain and the stem-anchor region [98]. Our partial DNA sequences of the *E* gene only covered the part of the E protein that includes part of the ectodomain, and the stem-anchor region. Our homology model of these partial sequences suggested that all non-synonymous substitutions of the Canton Valais isolates were located in the stem region of the E protein (Figure 4). The stem region plays an important role during the fusion process whereby the virus enters the target cells of the host [64, 98]. However, the functional role (if any) of these amino acid substitutions in the stem region of the E protein of TBEV remains unknown.

In Switzerland, the Federal Office of Public Health (FOPH) requires physicians and diagnostic laboratories to report all human cases of TBE. This mandatory reporting practice has been in effect in Switzerland since 1989 [99]. Over the last 11 years (2002 to 2013), the human caseload of TBE in Canton Valais (n = 14 cases) represents a tiny fraction (0.84%) of the total caseload in Switzerland (n = 1,668 cases). The human risk of TBE in Canton Valais is therefore low compared to other areas in Switzerland. However, the annual human caseload of TBE in Canton Valais has increased from 0.0 to 1.28 cases per 100,000 inhabitants over the last ten years (2002 to 2012), and over 78% (11/14) of the cases have occurred in the 5 last years. These observations suggest that TBE is a rising public health problem in Canton Valais. A similar increase in TBE has been observed in another alpine area in Central Europe, the state of Tyrol in Austria [100].

## Conclusions

In conclusion, this study confirms the presence of TBEV in *I. ricinus* tick populations in the southern part of Switzerland. In Canton Valais, the risk of TBEV appears to be highest along a 33 km stretch of the Rhône River between Salgesch and Brig. While the human caseload of TBE is low in Canton Valais it appears to be increasing. To limit the number of TBEV-infected patients in the future, we recommend educating the Valaisian population about the prevention of tick bites and the availability of the TBEV vaccine. Other methods of TBEV detection and surveillance should be used including seroepidemiological surveys of sentinel vertebrate hosts such as wild rodents or livestock.

## Electronic supplementary material

Additional file 1: **The 45 sites in Canton Valais, Switzerland that were sampled for** ***Ixodes ricinus*** **ticks.** For each site, the site name, year of sampling, altitude, and the GPS coordinates are shown. (DOCX 94 KB)

Additional file 2: **Primers and probes used in the qPCR to detect TBEV in** ***Ixodes ricinus*** **ticks.** The TBEV detection primers amplified an 87 bp segment of the *envelope* gene. The primers for the Mengo virus amplified a 103 bp segment of the 5’ non-coding region and were used as an internal control to confirm that each RT-qPCR reaction worked. (DOCX 66 KB)

Additional file 3: **The 55 TBEV sequences used in the phylogenetic analysis.** Shown are the TBEV subtype classification (Far-Eastern, Siberian, or European), the isolate name, the location where the isolate was obtained, and the Genbank accession number. (DOCX 93 KB)

Additional file 4: **Probability to detect at least one TBEV-positive tick for each combination of site and year.** The probability to detect at least one TBEV-positive tick (P_detect_) depends on the sampling effort for each combination of site and year. The sampling effort was based on the number of adults, nymphs, and larvae that were collected for each combination of site and year. The power analysis assumed that >1% of the ticks were infected with the tick-borne encephalitis virus. (DOCX 91 KB)

Additional file 5: **The PubMed accession number of the partial sequences of the NS5 gene and the** ***envelope*** **gene are shown.** These sequences came from the 28 pools of *Ixodes ricinus* ticks that tested positive for tick-borne encephalitis virus. These ticks had been collected from different sites in Canton Valais between 2010-2013. Pool content refers to whether the pools contained adult ticks, nymphal ticks, or a mixture. Also shown are the numbers of cycles at which the sample tested positive for each of the three replicate runs of the qPCR assay. (DOCX 102 KB)
