# Supplementary material for: Prevalence and phylogenetic analysis of tick-borne encephalitis virus (TBEV) in field-collected ticks (Ixodes ricinus) in southern Switzerland
Source: Parasit Vectors. 2014 Sep 22;7:443. doi: 10.1186/1756-3305-7-443 (PMC4261884; doi:10.1186/1756-3305-7-443)
Supplement: Supplementary file 1 — Additional file 1: The 45 sites in Canton Valais, Switzerland that were sampled for Ixodes ricinus ticks. For each site, the site name, year of sampling, altitude, and the GPS coordinates are shown. (DOCX 94 KB) [file 13071_2014_1622_MOESM1_ESM.docx]

Additional file 1: **The 45 sites in Canton Valais, Switzerland that were sampled for *Ixodes ricinus* ticks.** For each site, the site name, year of sampling, altitude (meters above sea level), and the GPS coordinates of the latitude and the longitude are shown.

| Site | Year | Altitude | Latitude | Longitude |
| --- | --- | --- | --- | --- |
| Agarn | 2010 | 800 | 46°17’23’’N | 7°40’24’’E |
| Bex | 2010 | 480 | 46°15’32’’N | 7°01’41’’E |
| Bramois | 2010 | 640 | 46°13’19’’N | 7°24’25’’E |
| Brigerbad | 2011 | 860 | 46°18’13’’N | 7°55’25’’E |
| Brig | 2013 | 700 | 46°19’27’’N | 8°00’26’’E |
| Holzji-Brig | 2011, 2013 | 800 | 46°18’18’’N | 7°58’44’’E |
| Champex d’Alesse-Dorénaz | 2010 | 1100 | 46°08’26’’N | 7°03’45’’E |
| Dorénaz | 2010 | 460 | 46°08’33’’N | 7°02’52’’E |
| Ergisch | 2011 | 840 | 46°17’24’’N | 7°42’19’’E |
| Ernen | 2011 | 1360 | 46°23’41’’N | 8°08’51’’E |
| Fiesch | 2011 | 1090 | 46°24’37’’N | 8°08’32’’E |
| La Douay-Orsières | 2010 | 820 | 46°03’02’’N | 7°09’02’’E |
| La Léchère-Finhaut | 2013 | 1400 | 46°04’38’’N | 6°57’50’’E |
| Le Trétien | 2012 | 1100 | 46°06’03’’N | 6°59’44’’E |
| Lerch-Unterbäch | 2011 | 790 | 46°18’01’’N | 7°47’48’’E |
| Les Crêts- Finhaut | 2013 | 1340 | 46°05’13’’N | 6°59’06’’E |
| Lufu- Niedergesteln | 2011, 2012 | 680 | 46°18’15’’N | 7°46’38’’E |
| Montagnon-Leytron | 2012 | 840 | 46°11’19’’N | 7°11’14’’E |
| Mörel | 2011 | 770 | 46°20’57’’N | 8°02’25’’E |
| Mt Chemin- Martigny | 2010 | 500 | 46°05’46’’N | 7°04’53’’E |
| Mt d’Ottan- Martigny | 2010, 2012 | 460 | 46°06’53’’N | 7°03’11’’E |
| Muraz | 2010 | 390 | 46°17’10’’N | 6°54’57’’E |
| Oberi Albe-Visp | 2011 | 890 | 46°17’43’’N | 7°51’28’’E |
| Oberlufu-Niedergesteln | 2011 | 700 | 46°18’06’’N | 7°47’22’’E |
| Ollon | 2010 | 640 | 46°17’52’’N | 7°00’13’’E |
| Pletschen-Susten | 2013 | 810 | 46°17’40’’N | 7°38’15’’E |
| Pramagon-Grône | 2010 | 940 | 46°14’32’’N | 7°26’56’’E |
| Raron | 2010 - 2013 | 640 | 46°17’56’’N | 7°48’46’’E |
| Riddes | 2010 | 520 | 46°10’55’’N | 7°14’59’’E |
| Rittergut-Visp | 2011, 2012 | 980 | 46°17’35’’N | 7°50’40’’E |
| Rossegga-Naters | 2013 | 870 | 46°19’26’’N | 7°58’52’’E |
| Salgesch | 2010 - 2013 | 570 | 46°17’53’’N | 7°34’44’’E |
| Salins | 2010 | 1060 | 46°12’25’’N | 7°21’38’’E |
| Sarreyer-Bagnes | 2011 | 1600 | 46°03’52’’N | 7°15’55’’E |
| Sembrancher | 2010 | 900 | 46°04’42’’N | 7°10’37’’E |
| Sion | 2010 | 640 | 46°13’59’’N | 7°20’15’’E |
| St-Léonard | 2010 | 540 | 46°15’53’’N | 7°25’04’’E |
| St-Maurice | 2010, 2012 | 490 | 46°11’33’’N | 7°00’54’’E |
| Stalden | 2011 | 980 | 46°13’28’’N | 7°52’11’’E |
| Steg | 2011 | 810 | 46°18’10’’N | 7°44’49’’E |
| Unterems | 2011 | 680 | 46°17’57’’N | 7°41’53’’E |
| Visp | 2011 | 740 | 46°17’26’’N | 7°53’27’’E |
| Vouvry | 2010 | 700 | 46°19’60’’N | 6°52’42’’E |
| Z’Brigg-Naters | 2013 | 700 | 46°19’11’’N | 7°58’41’’E |
| Zeneggen | 2011 | 820 | 46°17’08’’N | 7°52’34’’E |
