# Supplementary material for: Prevalence and phylogenetic analysis of tick-borne encephalitis virus (TBEV) in field-collected ticks (Ixodes ricinus) in southern Switzerland
Source: Parasit Vectors. 2014 Sep 22;7:443. doi: 10.1186/1756-3305-7-443 (PMC4261884; doi:10.1186/1756-3305-7-443)
Supplement: Supplementary file 2 — Additional file 2: Primers and probes used in the qPCR to detect TBEV in Ixodes ricinus ticks. The TBEV detection primers amplified an 87 bp segment of the envelope gene. The primers for the Mengo virus amplified a 103 bp segment of the 5’ non-coding region and were used as an internal control to confirm that each RT-qPCR reaction worked. (DOCX 66 KB) [file 13071_2014_1622_MOESM2_ESM.docx]

Additional file 2: **Primers and probes used in the qPCR to detect TBEV in *Ixodes ricinus* ticks.** The TBEV detection primers amplified an 87 bp segment of the *envelope* gene. The primers for the Mengo virus amplified a 103 bp segment of the 5’ non-coding region and were used as an internal control to confirm that each RT-qPCR reaction worked.

| Description of the target | Primer name | Sequence (5’→3’) | Position on gene | Product size (pb) |
| --- | --- | --- | --- | --- |
| TBEV, *envelope* gene |  |  |  | 87 |
| Forward | tbeE-F6 | GGCTTGTGAGGCAAAAAAGAA | 1329–1349 |  |
| Reverse | tbeE-R2 | TCCCGTGTGTGGTTCGACTT | 1397–1416 |  |
| Probe | TBEE-P4 | FAM-AAGCCACAGGACATGTGTACGACGCC | 1349–1374 |  |
|  |  |  |  |  |
| Mengovirus vMC_0_, 5’ non-coding region |  |  |  | 103 |
| Forward | Mengo-F1 | GACTACCCACTCCCCCTTTC | 64–83 |  |
| Reverse | Mengo-R1 | GCTTCGGCCAGTAATGTGAT | 147–166 |  |
| Probe | Mengo-P1 | JOE-TGAAGGCTACGATAGTGCCAGGGC | 88–111 |  |
|  |  |  |  |  |
