# Supplementary material for: Prevalence and phylogenetic analysis of tick-borne encephalitis virus (TBEV) in field-collected ticks (Ixodes ricinus) in southern Switzerland
Source: Parasit Vectors. 2014 Sep 22;7:443. doi: 10.1186/1756-3305-7-443 (PMC4261884; doi:10.1186/1756-3305-7-443)
Supplement: Supplementary file 3 — Additional file 3: The 55 TBEV sequences used in the phylogenetic analysis. Shown are the TBEV subtype classification (Far-Eastern, Siberian, or European), the isolate name, the location where the isolate was obtained, and the Genbank accession number. (DOCX 93 KB) [file 13071_2014_1622_MOESM3_ESM.docx]

Additional file 3: **The 55 TBEV sequences used in the phylogenetic analysis.** Shown are the TBEV subtype classification (Far-Eastern, Siberian, or European), the isolate name, the location where the isolate was obtained, and the Genbank accession number.

| Subtype | Isolate Name | Location | Genbank |
| --- | --- | --- | --- |
| Far-Eastern | 886-84 ^a^ | Russia | EF469662 |
|  | Senzhang ^d^ | China | AY182009 |
|  | Sofjin-HO ^d^ | Russia | AB062064 |
| Siberian | Zausaev ^d^ | Russia | AF527415 |
|  | Vasilchenko ^a^ | Siberia | AF069066 |
|  | Kokkola-86 ^b^ | Finland | DQ451307 |
| European | Greek goat encephalitis ^d^ | Greece | DQ235153 |
|  | Turkish sheep encephalitis ^d^ | Turkey | DQ235151 |
|  | K23 ^d^ | Germany | AM600965 |
|  | Toro-2003 ^a^ | Sweden | DQ401140 |
|  | 263 ^d^ | Czech Republic | U27491 |
|  | Hypr ^d^ | Czech Republic | U39292 |
|  | Neudoerfl ^d^ | Austria | U27495 |
|  | Est3476 ^d^ | Estonia | GU183383 |
|  | FVG ML Raccolana ^b^ | Italy | FJ159003 |
|  | FVG BM Forni di Sotto ^b^ | Italy | FJ159002 |
|  | Kumlinge A52 ^b^ | Finland | AJ298321 |
|  | Kamnik ^b^ | Slovenia | EU057641 |
|  | Stefanja gora ^b^ | Slovenia | EU057639 |
|  | LithT418 ^b^ | Lithuania | DQ112088 |
|  | NETBE1 ^b^ | Switzerland | HM450136 |
|  | NETBE2 ^b^ | Switzerland | HM450137 |
|  | NETBE3 ^b^ | Switzerland | HM450138 |
|  | NETBE4 ^b^ | Switzerland | HM450139 |
|  | NETBE5 ^b^ | Switzerland | HM450140 |
|  | NETBE6 ^b^ | Switzerland | HM450141 |
|  | NETBE7 ^b^ | Switzerland | HQ883372 |
|  | NETBE8 ^b^ | Switzerland | HQ883373 |
|  | NETBE9 ^b^ | Switzerland | HQ883374 |
|  | NETBE10 ^b^ | Switzerland | HQ883375 |
|  | NETBE11 ^b^ | Switzerland | HQ883376 |
|  | AG Brittnau 1 ^c^ | Switzerland | HM468123 |
|  | LU Dagmarsellen 1 ^c^ | Switzerland | HM468142 |
|  | UR Sisikon 1 ^c^ | Switzerland | HM468171 |
|  | TG Lommis 1 ^c^ | Switzerland | HM468158 |
|  | VS Raron ^c^ | Switzerland | HM468176 |
|  | VS Salgesch ^c^ | Switzerland | HM468177 |
|  | VD Rances ^c^ | Switzerland | HM468174 |
|  | ZH Langnau a.A.1 ^c^ | Switzerland | HM468187 |
|  | TG Thundorf ^c^ | Switzerland | HM468161 |
|  | AG Gipf-Oberfrick ^c^ | Switzerland | HM468128 |
|  | SH Stein am Rhein ^c^ | Switzerland | HM468149 |
|  | TG Wängi 1 ^c^ | Switzerland | HM468162 |
|  | ZH Bassersdorf 1 ^c^ | Switzerland | HM468184 |
|  | BE Belp ^c^ | Switzerland | HM468131 |
|  | BE Thun ^c^ | Switzerland | HM468141 |
|  | ZG Steinhausen 1 ^c^ | Switzerland | HM468178 |
|  | ZH Unterengstringen ^c^ | Switzerland | HM468192 |
|  | SO Oensingen 1 ^c^ | Switzerland | HM468150 |
|  | OW Alpnach ^c^ | Switzerland | HM468146 |
|  | LU Ebikon ^c^ | Switzerland | HM468145 |
|  | SZ Freienbach 1 ^c^ | Switzerland | HM468153 |
|  | ZH RütiZH ^c^ | Switzerland | HM468191 |
|  | SG Mörschwil 1 ^c^ | Switzerland | HM468147 |
|  | SZ Gersau ^c^ | Switzerland | HM468155 |

^a^ Complete genome available on Genbank

^b^ *NS5* gene available

^c^ *Envelope* gene available

^d^ Complete coding domain sequence (CDS)
