# Supplementary material for: Prevalence and phylogenetic analysis of tick-borne encephalitis virus (TBEV) in field-collected ticks (Ixodes ricinus) in southern Switzerland
Source: Parasit Vectors. 2014 Sep 22;7:443. doi: 10.1186/1756-3305-7-443 (PMC4261884; doi:10.1186/1756-3305-7-443)
Supplement: Supplementary file 4 — Additional file 4: Probability to detect at least one TBEV-positive tick for each combination of site and year. The probability to detect at least one TBEV-positive tick (Pdetect) depends on the sampling effort for each combination of site and year. The sampling effort was based on the number of adults, nymphs, and larvae that were collected for each combination of site and year. The power analysis assumed that >1% of the ticks were infected with the tick-borne encephalitis virus. (DOCX 91 KB) [file 13071_2014_1622_MOESM4_ESM.docx]

Additional file 4: **Probability to detect at least one TBEV-positive tick for each combination of site and year.** The probability to detect at least one TBEV-positive tick (P_detect_) depends on the sampling effort for each combination of site and year. The sampling effort was based on the number of adults, nymphs, and larvae that were collected for each combination of site and year. The power analysis assumed that >1% of the ticks were infected with the tick-borne encephalitis virus.

| Date | Site | Adults | Nymphs | Larvae | Total | Positive pools/  total pools | P_detect_ |
| --- | --- | --- | --- | --- | --- | --- | --- |
| 21.06.2010 | Agarn | 71 | 233 | 0 | 304 | 0/8 | 0.95 |
| 01.07.2010 | Bex | 54 | 199 | 0 | 253 | 0/7 | 0.92 |
| 23-25.06.10 | Bramois | 183 | 84 | 0 | 267 | 0/11 | 0.93 |
| 07.07.2010 | Champex d’Allesse-Dorénaz | 119 | 114 | 0 | 233 | 0/9 | 0.90 |
| 17.06-02.07.10 | Dorénaz | 54 | 16 | 0 | 70 | 0/4 | 0.51 |
| 09.07.2010 | La Douay- Orsières | 217 | 154 | 0 | 371 | 0/15 | 0.98 |
| 25.05.2010 | Mt Chemin-Martigny | 269 | 287 | 0 | 556 | 0/19 | 1.00 |
| 25.05.2010 | Mt d’Ottan-Martigny | 101 | 132 | 0 | 233 | 0/8 | 0.90 |
| 26.06.2010 | Muraz | 123 | 120 | 0 | 243 | 0/8 | 0.91 |
| 12.07.2010 | Ollon | 83 | 145 | 0 | 228 | 0/7 | 0.90 |
| 08.07.2010 | Pramagon-Grône | 15 | 28 | 0 | 43 | 0/2 | 0.35 |
| 07.05-14.06.10 | Raron | 875 | 872 | 2 | 1749 | 13/201 | 1.00 |
| 02.07.2010 | Riddes | 10 | 4 | 0 | 14 | 0/2 | 0.13 |
| 14-22.06.10 | Salgesch | 271 | 218 | 0 | 489 | 1/17 | 0.99 |
| 15.07.2010 | Salins | 73 | 30 | 0 | 103 | 0/5 | 0.64 |
| 05.07.2010 | Sembrancher | 141 | 151 | 0 | 292 | 0/10 | 0.95 |
| 15.06.2010 | Sion | 212 | 28 | 0 | 240 | 0/10 | 0.91 |
| 30.06.2010 | St-Léonard | 96 | 1 | 0 | 97 | 0/5 | 0.62 |
| 29.06.2010 | St-Maurice | 109 | 373 | 0 | 482 | 0/14 | 0.99 |
| 24.06.2010 | Vouvry | 123 | 117 | 0 | 240 | 0/8 | 0.91 |
|  | Total 2010 | 3199 | 3306 | 2 | 6507 | 14/370 |  |
|  |  | 49.16% | 50.81% | 0.03% |  |  |  |
| 05.05.2011 | Brigerbad | 10 | 1 | 0 | 11 | 0/3 | 0.10 |
| 18.05.2011 | Ergisch | 62 | 206 | 0 | 268 | 0/10 | 0.93 |
| 06.05.2011 | Ernen | 0 | 25 | 0 | 25 | 0/1 | 0.22 |
| 15-21.06.2011 | Fiesch | 268 | 314 | 0 | 582 | 0/32 | 1.00 |
| 02-10.05.2011 | Holzji-Brig | 75 | 478 | 2 | 555 | 0/18 | 1.00 |
| 17.05.2011 | Lerch-Unterbäch | 132 | 355 | 0 | 487 | 0/22 | 0.99 |
| 17.05.2011 | Lufu-Niedergesteln | 38 | 66 | 0 | 104 | 2/4 | 0.65 |
| 14-24.06.2011 | Mörel | 443 | 80 | 0 | 523 | 0/47 | 0.99 |
| 20.05.2011 | Oberi Albe-Visp | 229 | 419 | 0 | 648 | 0/38 | 1.00 |
| 18.05.2012 | Oberlufu-Niedergesteln | 85 | 40 | 0 | 125 | 0/9 | 0.72 |
| 06.04.2011 | Raron | 156 | 124 | 0 | 280 | 2/38 | 0.94 |
| 20.05-12.07 2011 | Rittergut-Visp | 73 | 10 | 0 | 83 | 2/7 | 0.57 |
| 18.04-10.06.2011 | Salgesch | 320 | 1012 | 0 | 1332 | 3/61 | 1.00 |
| 16.06.2011 | Sarreyer-Bagnes | 6 | 27 | 0 | 33 | 0/1 | 0.28 |
| 27.06.2011 | Stalden | 4 | 85 | 1 | 90 | 0/3 | 0.60 |
| 27.04.2011 | Steg | 160 | 554 | 1 | 715 | 0/26 | 1.00 |
| 19.05.2011 | Unterems | 79 | 501 | 3 | 583 | 0/16 | 1.00 |
| 26.04.2011 | Visp | 118 | 232 | 0 | 350 | 0/17 | 0.97 |
| 21.05.2011 | Zeneggen | 4 | 6 | 0 | 10 | 0/1 | 0.10 |
|  | Total 2011 | 2262 | 4535 | 7 | 6804 | 9/354 |  |
|  |  | 33.25% | 66.65% | 0.10% |  |  |  |
| 19.10.2012 | Le Trétien | 10 | 201 | 1 | 212 | 0/7 | 0.88 |
| 07.05-28.06.12 | Lufu-Niedergesteln | 62 | 24 | 0 | 86 | 0/5 | 0.58 |
| 18.10.2012 | Montagnon-Leytron | 4 | 69 | 0 | 73 | 0/4 | 0.52 |
| 27.03- 24.10.12 | Mt d’Ottan- Martigny | 644 | 83 | 0 | 727 | 0/57 | 1.00 |
| 27.03- 24.10.12 | Raron | 572 | 143 | 3 | 718 | 0/51 | 1.00 |
| 07.05-28.06.12 | Rittergut-Visp | 22 | 104 | 2 | 128 | 0/4 | 0.72 |
| 27.03- 24.10.12 | Salgesch | 88 | 379 | 0 | 467 | 0/32 | 0.99 |
| 27.03- 24.10.12 | St-Maurice | 78 | 324 | 0 | 402 | 0/22 | 0.98 |
|  | Total 2012 | 1480 | 1327 | 6 | 2813 | 0/182 |  |
|  |  | 52.61% | 47.17% | 0.21% |  |  |  |
| 17.03-08.05.13 | Brig | 397 | 213 | 1 | 611 | 1/24 | 1.00 |
| 08.05.2013 | Holzji-Brig | 4 | 19 | 0 | 23 | 0/1 | 0.21 |
| 14.05-26.06.13 | La Léchère-Finhaut | 73 | 387 | 1 | 461 | 0/14 | 0.99 |
| 06.06-27.06.13 | Les Crêts- Finhaut | 28 | 585 | 189 | 802 | 0/24 | 1.00 |
| 25.06.2013 | Pletschen-Susten | 7 | 2 | 0 | 9 | 1/1 | 0.09 |
| 17.04.2013 | Raron | 261 | 239 | 0 | 500 | 1/21 | 0.99 |
| 28.05-03.06.13 | Rossegga-Naters | 9 | 52 | 0 | 61 | 0/15 | 0.46 |
| 04.06-11.06.13 | Salgesch | 222 | 434 | 6 | 662 | 2/24 | 1.00 |
| 03.06.2013 | Z’Brigg-Naters | 34 | 43 | 1 | 78 | 0/3 | 0.54 |
|  | Total 2013 | 1035 | 1974 | 198 | 3207 | 5/127 |  |
|  |  | 32.27% | 61.55% | 6.17% |  |  |  |
|  | Total 2010-2013 | 7976 | 11142 | 213 | 19331 | 28/1033 |  |
|  |  | 41.26% | 57.64% | 1.10% |  | 2.71% |  |
