# Supplementary material for: Prevalence and phylogenetic analysis of tick-borne encephalitis virus (TBEV) in field-collected ticks (Ixodes ricinus) in southern Switzerland
Source: Parasit Vectors. 2014 Sep 22;7:443. doi: 10.1186/1756-3305-7-443 (PMC4261884; doi:10.1186/1756-3305-7-443)
Supplement: Supplementary file 5 — Additional file 5: The PubMed accession number of the partial sequences of the NS5 gene and the envelope gene are shown. These sequences came from the 28 pools of Ixodes ricinus ticks that tested positive for tick-borne encephalitis virus. These ticks had been collected from different sites in Canton Valais between 2010-2013. Pool content refers to whether the pools contained adult ticks, nymphal ticks, or a mixture. Also shown are the numbers of cycles at which the sample tested positive for each of the three replicate runs of the qPCR assay. (DOCX 102 KB) [file 13071_2014_1622_MOESM5_ESM.docx]

Additional file 5: **The PubMed accession number of the partial sequences of the NS5 gene and the *envelope* gene are shown.** These sequences came from the 28 pools of *Ixodes ricinus* ticks that tested positive for tick-borne encephalitis virus. These ticks had been collected from different sites in Canton Valais between 2010-2013. Pool content refers to whether the pools contained adult ticks, nymphal ticks, or a mixture. Also shown are the numbers of cycles at which the sample tested positive for each of the three replicate runs of the qPCR assay.

|  |  |  | qPCR result for three runs | | | Pubmed accession number | |
| --- | --- | --- | --- | --- | --- | --- | --- |
| Site | Year | Pool content | 1 | 2 | 3 | *NS5* gene | *Envelope* gene |
| Raron | 2010 | Adults | 22.83 | 23.05 | 23.97 | KF819427 ^a^ | KF819406 ^b^ |
| Raron | 2010 | Adults | 30.53 | 26.54 | 27.36 | KF819428 ^a^ | KF819407 ^b^ |
| Raron | 2010 | Adults | 31.09 | 30.26 | 31.69 | NA | NA |
| Raron | 2010 | Adults | 37.59 | 37.01 | 38.03 | NA | NA |
| Raron | 2010 | Adults | 33.70 | 33.21 | 34.08 | NA | NA |
| Raron | 2010 | Adults | 26.39 | 28.06 | 28.84 | KF819429 ^a^ | NA |
| Raron | 2010 | Adults | 17.93 | 21.77 | 22.90 | KF819430 ^a^ | KF819408 ^b^ |
| Raron | 2010 | Mixture | 27.02 | 30.09 | 31.08 | KF819431 ^a^ | NA |
| Raron | 2010 | Adults | 22.71 | 22.82 | 23.74 | KF819432 ^a^ | KF819409 ^b^ |
| Raron | 2010 | Mixture | 25.78 | 23.72 | 24.67 | KF819433 ^a^ | KF819410 ^b^ |
| Raron | 2010 | Adults | 21.63 | 22.65 | 23.68 | KF819434 ^a^ | KF819411 ^b^ |
| Raron | 2010 | Adults | 27.51 | 31.21 | 32.24 | KF819435 ^a^ | NA |
| Raron | 2010 | Adults | 19.01 | 15.68 | 17.93 | KF819436 ^a^ | KF819412 ^b^ |
| Salgesch | 2010 | Nymphs | 25.78 | 25.15 | 25.96 | KF819437 ^a^ | KF819413 ^b^ |
| Raron | 2011 | Adults | 25.81 | 25.63 | 25.25 | KF819438 ^a^ | KF819414 ^b^ |
| Raron | 2011 | Adults | 28.15 | 27.84 | 28.43 | KF819439 ^a^ | KF819415 ^b^ |
| Salgesch | 2011 | Nymphs | 31.29 | 31.97 | 31.17 | KF819440 ^a^ | KF819416 ^b^ |
| Salgesch | 2011 | Adults | 25.90 | 25.90 | 24.79 | KF819441 ^a^ | KF819417 ^b^ |
| Salgesch | 2011 | Nymphs | 29.10 | 29.52 | 29.30 | KF819442 ^a^ | NA |
| Rittergut-Visp | 2011 | Adults | 31.51 | 29.01 | 29.76 | KF819443 ^a^ | KF819418 ^b^ |
| Rittergut-Visp | 2011 | Adults | 29.04 | 30.02 | 29.40 | KF819444 ^a^ | KF819419 ^b^ |
| *Lufu-Nieder | 2011 | Adults | 27.27 | 28.76 | 27.85 | KF819445 ^a^ | KF819420 ^b^ |
| *Lufu-Nieder | 2011 | Nymphs | 25.43 | 27.16 | 26.56 | KF819446 ^a^ | KF819421 ^b^ |
| Raron | 2013 | Mixture | 23.91 | 25.12 | 24.60 | KF819447^b^ | KF819422 ^b^ |
| Salgesch | 2013 | Nymphs | 30.31 | 31.48 | 31.76 | KF819448 ^b^ | KF819423 ^b^ |
| Salgesch | 2013 | Adults | 28.15 | 28.85 | 28.40 | KF819449 ^b^ | KF819424 ^b^ |
| Brig | 2013 | Adults | 33.93 | 33.15 | 33.71 | KF819450 ^b^ | KF819425 ^b^ |
| Pletschen-Susten | 2013 | Mixture | 30.41 | 31.12 | 30.80 | KF819451 ^b^ | KF819426 ^b^ |
| Total |  |  | 28 TBEV-positive samples | | | 25 sequences | 21 sequences |

^a^ Sequenced with the in-house protocol

^b^ Sequenced by Microsynth

NA Sequences not available

* Full name of this site is Lufu-Niedergesteln
